# Devitalizing Pulps for Crowns

**Published:** 1918-06

**Authors:** H. J. Groslee


### DEVITALIZING PULPS FOR CROWNS

BY H. J. GROSLEE, D.D.S.

About eighteen years ago I read a paper before the Chicago Dental Society in which I took the position that the pulps of teeth which were to be prepared for the reception of gold crowns should almost invariably be devitalized, with certain exceptions duly noted. I want to say now that I did not mean at that time that this procedure should be followed because it was easier to do it that way, or because of the fact that the pulp had no physiological value whatever, or because a tooth with a dead pulp in it would be better than a tooth with a live pulp in it. Not at all. But what occasioned my remarks and caused me to take the stand I took was this: it had been my observation that it was quite—if not, indeed, almost—impossible for me to give to a tooth, a typical molar tooth, for example, the necessary mechanical preparation which would permit me, or make it *possible* for me to properly and more or less accurately fit that tooth with a telescoping band if it had a vital pulp in it. Hence, I took the stand that, since irritation of a mechanical nature must result if the band did not fit, and since it was absolutely necessary, therefore, to make the band fit, that in order to have a band which did fit if it became necessary for mechanical reasons to devitalize the pulp in order to enable me to do it, that is what should be done, and I am still of the same opinion today. Of course, we should conserve the vitality of the pulp of every tooth whenever and wherever it is possible, but I will also submit that if we need to use that tooth, and if that tooth demands restoration, and then if *adaptation* is one of the requirements of success and permanency in the restoration, whatever may be the type of restoration used, and if in turn it is necessary to devitalize the pulp in order to effect and obtain that adaptation, then to do it rather than to leave the pulp alive at the expense of correct adaptation is the better practice. This has to do with the absolute mechanical requirements of peripheral adaptation in the preparation of teeth for the reception of gold shell crowns.

I now want to ask you this question: If it is thus practically impossible to prepare a typical tooth for the reception of a gold shell crown such as we now make, when the tooth has a vital pulp in it, is it possible to give to teeth such further peripheral preparation as the shoulder crown demands, and still conserve the pulp? That the shoulder crown is a beautiful form of mechanical adaptation there is no question. Its application affords a means of obtaining a continuity between the artificial restoration and the supporting root, and, as that continuity may be made more or less perfect, in that same proportion will the degree of irritation become less and less. If it is true that it is usually quite impossible to prepare a vital tooth for the reception of a telescoping band which fits over the outside of it, then how can we cut enough more to form a shoulder and thus obtain a more perfect continuity in the adaptation of the crown to the root? It is possible that in Minneapolis, under ideal conditions, this may be done, but, generally speaking, I have not been able to do it, and I do not believe the conditions are any different today than they have been at any time since I have been in the practice of dentistry. We all want to save the pulps of teeth if we can. It is our duty to do so. Nobody, I think, in this audience would question that a tooth with a vital pulp in it is a better tooth than a tooth with a dead pulp in it; but if it becomes a question of whether we can utilize a tooth with a dead pulp in it to better advantage than we can by conserving its vitality, which of the two routes should we take? Our signal ambition in the decision which we must make should be the degree of permanency, usefulness and comfort which will obtain, and this will always be a question largely, if not entirely, of judgment on our part, and judgment can only come from experience. —*Dental Review*.

